# Transcriptomic analysis reveals protein homeostasis breakdown in the coral *Acropora millepora* during hypo-saline stress

**DOI:** 10.1186/s12864-019-5527-2

**Published:** 2019-02-20

**Authors:** Catalina Aguilar, Jean-Baptiste Raina, Sylvain Fôret, David C. Hayward, Bruno Lapeyre, David G. Bourne, David J. Miller

**Affiliations:** 1 AIMS@JCU and Department of Molecular and Cell Biology, James Cook University, Townsville, Queensland 4811 Australia; 2 ARC Centre of Excellence for Coral Reef Studies and Department of Molecular and Cell Biology, James Cook University, Townsville, Queensland 4811 Australia; 3 Cooperative Institute for Marine and Atmospheric Studies, Rosenstiel School of Marine & Atmospheric Science, University of Miami, 4600 Rickenbacker Causeway, Miami, Florida 33149 USA; 4 Atlantic Oceanographic and Meteorological Laboratories (AOML), NOAA, 4301 Rickenbacker Causeway, Miami, Florida 33149 USA; 5 Climate Change Cluster (C3), University of Technology, Sydney, NSW 2007 Australia; 6 Division of Ecology and Evolution, Research School of Biology, Australian National University, Canberra, ACT 2601 Australia; 7 Laboratoire d’excellence CORAIL, Centre de Recherches Insulaires et Observatoire de l’Environnement (CRIOBE), Moorea, B.P.1013, Papeete French Polynesia; 8 Australian Institute of Marine Science, Townsville, Queensland 4810 Australia; 9 College of Science and Engineering, James Cook University, Townsville, 4811 Australia

**Keywords:** Coral, Transcriptomics, Salinity stress, Endoplasmic reticulum, Amino acid metabolism

## Abstract

**Background:**

Coral reefs can experience salinity fluctuations due to rainfall and runoff; these events can have major impacts on the corals and lead to bleaching and mortality. On the Great Barrier Reef (GBR), low salinity events, which occur during summer seasons and can involve salinity dropping ~ 10 PSU correlate with declines in coral cover, and these events are predicted to increase in frequency and severity under future climate change scenarios. In other marine invertebrates, exposure to low salinity causes increased expression of genes involved in proteolysis, responses to oxidative stress, and membrane transport, but the effects that changes in salinity have on corals have so far received only limited attention. To better understand the coral response to hypo-osmotic stress, here we investigated the transcriptomic response of the coral *Acropora millepora* in both adult and juvenile life stages to acute (1 h) and more prolonged (24 h) exposure to low salinity.

**Results:**

Differential gene expression analysis revealed the involvement of both common and specific response mechanisms in *Acropora*. The general response to environmental stressors included up-regulation of genes involved in the mitigation of macromolecular and oxidative damage, while up-regulation of genes involved in amino acid metabolism and transport represent specific responses to salinity stress.

**Conclusions:**

This study is the first comprehensive transcriptomic analysis of the coral response to low salinity stress and provides important insights into the likely consequences of heavy rainfall and runoff events on coral reefs.

**Electronic supplementary material:**

The online version of this article (10.1186/s12864-019-5527-2) contains supplementary material, which is available to authorized users.

## Background

Coral reefs are amongst the most diverse and complex of ecosystems and, as well as their biological significance, are of enormous social and economic importance [1]. However, they are experiencing long-term decline on a global scale due to overfishing, pollution, and climate change [2, 3]. Climate change is likely to be an increasingly significant cause of coral loss through thermal stress and ocean acidification [4, 5], but also via increases in the frequency and intensity of tropical cyclones, which can expose coral reefs to more extreme and sudden salinity variations [6–8]. These conditions affect the Great Barrier Reef (GBR), where rain associated with tropical cyclones can lower the salinity of surface waters significantly (up to 10 PSU) [9], with these hypo-saline conditions sometimes prevailing for weeks [10]. Although the impacts of heavy rainfall can be correlated with coral decline on the GBR [11], the physiological effects of hypo-saline stress have not been thoroughly investigated. A few studies have described loss of *Symbiodiniaceae* and coral mortality following hypo-saline stress events [12–14], but no data are available on the molecular response of corals during these events.

Like many other marine invertebrates, corals are considered to be osmoconformers –their internal environment is near isotonic with the external environment – and can only tolerate a relatively narrow range of salinity (i.e. they are stenohaline). Our current understanding of osmoregulation processes in corals is largely derived from other marine invertebrates such as sea anemones and bivalves; in these organisms, small organic molecules and inorganic ions are used to prevent osmotic lysis [15, 16]. These molecules, known as osmolytes, include free amino acids (FAAs), FAA derivates (taurine, glycine betaine), floridoside and other compounds such as dimethylsulfoniopropionate (DMSP) [17, 18]. In many cases, organisms use a variety of osmolytes and related species may use quite different mechanisms. For example, the sea anemone *Metridium senile,* and marine sponges *Halichondria okadai* and *H. japonica* exhibit a general decrease of their FAA content during hypo-osmotic stress, whereas FAA content appears to increase in the coral *Acropora aspera* under these conditions [16, 19, 20]. Several other environmental stressors, such as temperature and elevated CO_2_, cause changes in the expression of specific molecular chaperones in corals [21, 22] and these are likely to be components of a general stress response system.

While the literature on the molecular responses of corals to hypo-osmotic stress is very limited, comprehensive datasets are available for some other marine invertebrates [21, 22]. In mussels, for example, responses to hypo-osmotic stress include increases in levels of oxidative stress proteins and proteolysis, as well as changes in expression of membrane transporter proteins, although closely related species have been shown to respond differently [23]. In the present study, the transcriptomic response of the common reef-building coral *Acropora millepora* to hypo-saline conditions was investigated. Through the availability of a whole genome assembly and a comprehensive set of protein predictions for this organism, it is now possible to compare the response of this coral to those of other marine invertebrates, to tease apart specific and general responses to different environmental stressors [21, 24, 25]. Here we exposed adult colonies of *Acropora millepora*, as well as aposymbiotic juveniles (devoid of any photosynthetic symbionts), to hypo-saline conditions mimicking those experienced in extreme weather events (25 PSU for the adults and 28 PSU for the juveniles). Although changes in the microbiome have previously been reported [26], this is the first study to comprehensively describe the molecular response of a coral to hypo-saline stress, and identifies both specific and general components of the response of *A. millepora* to environmental stressors.

## Methods

### Coral salinity stress experiment

The work described here was carried out under GBRMPA permit G09/30327.1. Eight *Acropora millepora* colonies were collected from Orpheus Island, Queensland, Australia (18°39′52. 43″S, 146°29′42.38″E) in June 2013 and transferred to the Australian Institute of Marine Science’s National Sea Simulator (SeaSim) facility where the colonies were acclimated for 14 days in outdoor aquaria at ~ 27 °C. Each colony was fragmented into 25 nubbins (~ 6 cm) that were then randomly distributed across nine 50 L tanks. The tanks were linked to a computer-controlled flow-through system supplying 0.4 μm filtered seawater (FSW) maintained at 25.7 °C (±0.6 °C) and an ambient salinity of 35 PSU. UV-filtered lights were mounted above each tank and nubbins were exposed to an intensity of 250 μE over a 12:12 h light/dark cycle (type of lights: 400 W metal halide lamps, BLV). The nubbins were acclimated in this system for a further 19 days to allow recovery. At the beginning of the experiment, corals were transferred to two tanks (one per treatment), the flow was stopped to ensure no water exchange and tanks were oxygenated via a pump (Tunze 6015). The nubbins were subsequently exposed to one of two salinity regimes for the duration of the experiment (24 h): ambient/control salinity of 35 PSU (*n* = 45), or low salinity of 25 PSU (*n* = 40). Some nubbins were used as test samples before the start of the experiment and to test the effects of treatments not included in this study, hence the larger number of nubbins at the beginning of the experiment. The 25 PSU FSW was prepared by diluting 700 ml of 35 PSU FSW with 300 ml reverse-osmosis water. The temperature during the treatment period was maintained at 25.9 ± 0.7 °C. Salinity was monitored using a water quality meter (TPS 90FL, ThermoFisher). Coral nubbins (*n* = 1 per colony, for both control and treatment) were sampled at two time points for RNA analysis, at 1 and 24 h post the salinity change (samples were taken at 12:00 and 1:00 pm respectively). A total of 18 nubbins for RNA analysis (*n* = 5 per time point for each condition, but *n* = 3 in the case of the 24 h treatment) were snap frozen in liquid nitrogen and stored at − 80 °C.

### Juvenile coral salinity stress experiment

For the experiment on coral juveniles, six *Acropora millepora* colonies were collected from Trunk Reef, GBR, Australia (18°22′15.10″S/ 146°48′27.82″E) and transferred to the National Sea Simulator (SeaSim) facility prior to the predicted spawning event in November 2013. Colonies were individually placed in 70 L tanks with 0.2 μm of filtered seawater (FSW). After spawning, gametes were collected and mixed to allow fertilization to occur. Fertilized embryos were transferred to larval rearing tanks and raised as described in Tebben et al. [27] and Raina et al. [28]. At 13 days post-fertilization, larvae were collected using a 1 mm mesh net, washed three times in 0.2 μm FSW and then settled in (sterile) 6-well plates (8 plates per species, 40 larvae per well; each well filled with 4 ml of ambient salinity (35 PSU) 0.2-μm FSW) using a cue (5 μL) derived from crustose coralline algae (CCA; see Siboni [29]). Throughout the incubation phase, the plates were maintained in the dark at 26.3 °C (± 0.01) and the FSW was changed every second day. Four days post-settlement (T0), plates were separated into two groups: 16 plates were maintained at 35 PSU (control salinity) while the seawater in the remaining 16 plates was exchanged for 28 PSU (salinity stress treatment). The salinity stress applied in the case of the juveniles (28 PSU) was slightly less challenging than that used for the adults (25 PSU) based on the high mortality rate observed at 25 PSU during a pilot study. Samples were collected for RNA after 24 h (T24), and 48 h (T48) at 2:00 pm each day (*n* = 23 samples; *n* = 6 wells per treatment per time point, but *n* = 5 in the case of the 48 h control).

### RNA extraction sequencing and gene expression analyses

Total RNA was extracted from 18 adult nubbins of five genotypes of 25 and 35 PSU treatments (n = 5 for each condition per time point, but *n* = 3 for 24 h treatment) following the same methods described in Aguilar et al. [30]. Coral juveniles were sampled by removing the water and adding 1.5 mL of RNA*later* (Ambion, cat# AM7021) simultaneously to each well (*n* = 40 juveniles) and scraping the content with a sterile 200 μL plastic tip to transfer the contents into a 2 mL tube and stored at − 20 **°**C. Twenty four samples (*n* = 6 wells per treatment per time point) from the content of each well were used to extract total RNA using the RNAaqueous-Micro total RNA isolation kit (AM1931, AMBION). The quality and quantity of RNA preparations were determined using a Bioanalyzer (Agilent 2100 Bioanalyzer) with samples prepared following the Agilent RNA 6000 Nano Kit instructions (cat # 5067–1511).

RNAseq libraries (18 for the adults and 23 for the juveniles) were constructed using the NEB Next Ultra Directional RNA Library Prep Kit for Illumina (NEB, E7420S) following the manufacturers recommended protocol, and 100 bp paired-end sequence data obtained using a HiSeq 2000 at the Biomolecular Resource Facility (Australian National University). After trimming (70–80% of reads retained), reads were mapped onto the *Acropora millepora* genome (Ying et al., in prep) using TopHat2 [31] and counts data generated using htseq-count [32] for subsequent analysis. The mapping efficiency varied significantly, in general being much higher for larvae (> 80%) than for adults (45–60%) due to the presence of Symbiodiniaceae in the latter. Approximately 70% of mapped reads were counted as genes. Genes IDs in this paper refer to the *A. millepora* protein predictions (e.g. 1.2.1.m1) that have been deposited in the Gene Expression Omnibus (GEO) under the reference number GSE96916.

The read count matrix was analysed in R (R Core Team 2014) using the package arrayQualityMetrics [33] to check for outliers, and was transformed (variance stabilizing transformation (VST)) to be visualized using a principal component analysis (PCA). Although potential effects of pseudoreplication caused by using only one tank per condition could not be tested, note that this has also frequently also been the case in similar published gene expression studies [34, 35]. The DESeq2 package [36] was used to test for differential gene expression due to the effects of salinity, while controlling for the effect of the genotype in the case of the adult dataset (design = ~ genotype + treatment). For the juvenile dataset, as each sample was a mixture of genotypes, genotype effects could not be considered (design = ~ treatment). Default functions for estimating size factors, dispersion and negative binomial Wald Test were used in DESeq2. Log2 fold changes (log2FC) in gene expression levels were obtained in DESeq2 by comparing control (35 PSU) vs. salinity treatment of four different comparisons: (i) control vs. treatment at 1 h in the adults, (ii) control vs. treatment at 24 h in the adults, (iii) control vs. treatment at 24 h in the juveniles and, (iv) control vs. treatment at 48 h in the juveniles. False discovery rate (FDR) adjusted *p* values were controlled at 5% for each gene according to the methods of Benjamini and Hochberg [37].

Statistically over-represented gene ontology (GO) categories were determined in BiNGO [38] in Cytoscape 3.1.1 [39] using the hypergeometric test and a FDR significance level of < 0.01 on the set of genes that were differentially up- or down-regulated in each dataset. These GO categories were used to search specific pathways in the Kyoto Encyclopedia of Genes and Genomes (KEGG) by downloading pathway sequences (using *Homo sapiens* and *Nematostella vectensis* as references for the pathways: protein processing in endoplasmic reticulum, nve0414, hsa04141; proteasome, nve03050, hsa03050; peroxisome, nve04146, hsa04146; lysosome, nve04142, hsa04142; glycine, serine and threonine metabolism, nve00260, hsa00260; glutathione metabolism, nve00480, hsa00480; alanine, aspartate and glutamate metabolism, nve00250, hsa00250; ABC transporters, nve02010, hsa02010) and blasting these sequences against the *A. millepora* protein predictions. All results are based on similarity of the *A. millepora* protein predictions to a reference annotated protein (evalue cut-off = 1e^− 4^).

## Results

### Differential gene expression analyses

**Fig. 1:**
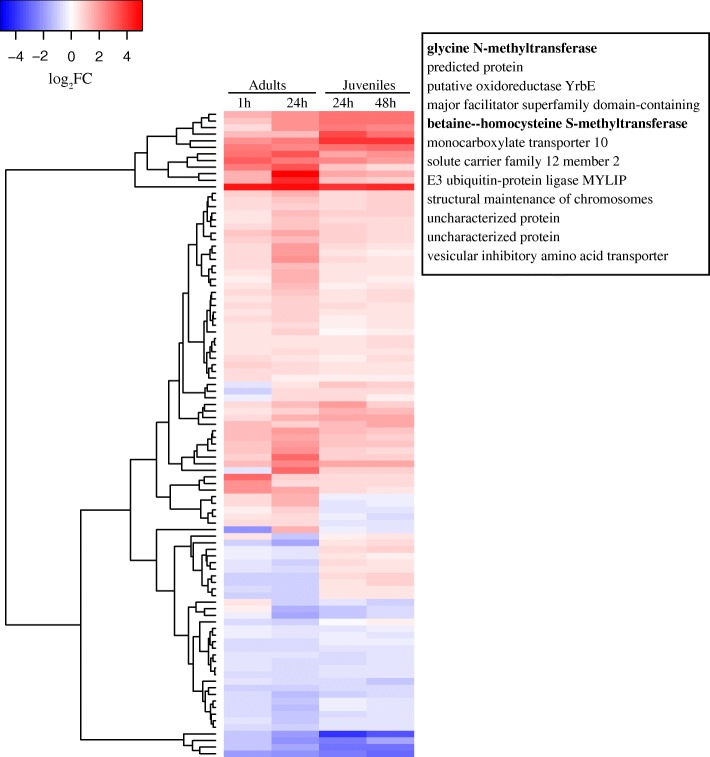
Heatmap (log_2_FC) summarising expression relative to 35 PSU controls of the suite of 98 genes which responded (FDR < 0.05) in all four salinity treatments. As indicated above the corresponding columns in the heatmap, the treatments were: 1 h (25 PSU) adults; 24 h (25 PSU) adults; 1 h (28 PSU) juveniles; 48 h (28 PSU) juveniles. The dendrogram on the left of the figure shows clustering based on similarity of expression pattern. To the right of the figure, the 12 most highly up-regulated genes are listed, those in bold being components of the free amino acid cycle (Additional file 4: Table S5). These 12 genes constitute a well resolved clade at the top of the heat map. For the complete list of genes IDs refer to Additional file 4: Table S1. The colour scale bar indicates up (red) or down-regulation (blue) relative to the control

Between 5.5–10.2 million RNAseq reads were obtained from each adult coral sample, while 3.4–8.8 million reads were recovered from each juvenile coral sample. PCA of the count matrix of the 26,622 *A. millepora* gene predictions revealed that the colony (i.e. genotype) had a stronger effect on gene expression than the salinity treatment for the adult corals, while in the case of juveniles (where genotype effects could not be accounted for) variation was primarily explained by treatment (Additional file 1: Figure S1). After 1 h of salinity stress, 2657 genes were differentially expressed in adults (DEGs; FDR < 0.05), increasing to 3713 after 24 h of exposure (Additional file 2: Figure S2). Whilst 3462 genes were differentially expressed in the juveniles after 24 h of salinity stress, this number decreased to 1485 after 48 h of stress (Additional file 2: Figure S2). At the 24 h time point, adults and juveniles shared 38% of up-regulated genes (total number: 1707; FDR < 0.05) and 31% of down-regulated genes (total number: 1755; FDR < 0.05; see Additional file 3: Figure S3). Moreover, the overall response to salinity stress included 98 genes that were differentially expressed in both adults and juveniles at both time points, amongst which were several genes involved in amino acid metabolism discussed below (Fig. 1, Additional file 4: Table S1).

Eighty-four GO terms were over-represented in the adults after 1 h of salinity stress, while this number decreased to 13 GO terms after 24 h (Additional file 4: Table S2). For the juveniles, 48 GO terms were over-represented after 24 h of salinity stress and this number decreased to 33 GO terms after 48 h. At both time points the GO term with the highest FDR was the ‘small molecular metabolic process’ (FDR 1.17E-12 and 5.24E-07 after 24 and 48 h). GO analysis revealed several categories that were down-regulated after 1 h but up-regulated after 24 h in the adults: (i) protein homeostasis, including: endoplasmic reticulum (ER), ER lumen, proteasome complex, cell catabolism and oxidoreductase activity; and (ii) amino acid and nitrogen metabolism. Based on these results, we used the sequences from specific KEGG pathways to find homologues in the coral transcriptome and understand the responses of these genes to hypo-saline stress.

### Proteolysis within the ER under hypo-saline conditions

**Fig. 2:**
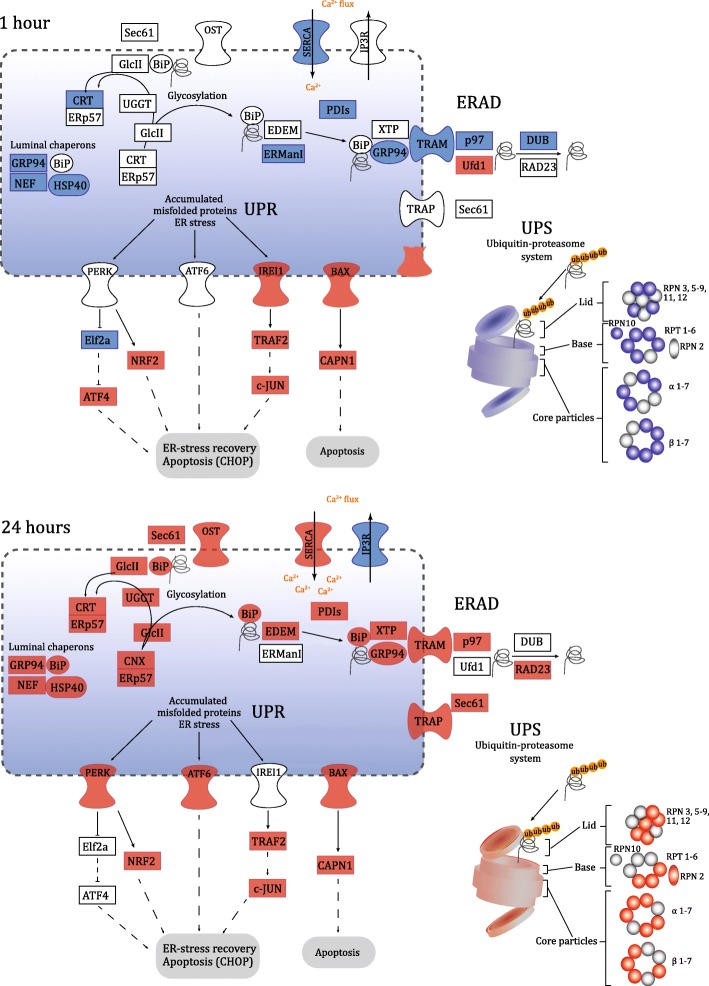
Differential expression of *A. millepora* homologues of components of the ER protein processing machinery (pathway 04141) after exposure of adult corals to 1 and 24 h of hypo-saline conditions. Colours represent genes (FDR < 0.05) that are up (red) or down-regulated (blue). The systems involved in ER protein processing and ER stress are indicated: glycosylation, ER associated degradation (ERAD), ubiquitin-proteasome system (UPS), and the unfolded protein response (UPR). A complete list of the genes involved in this pathway and log_2_FC values is provided as Additional file 4: Table S3. Figure adapted from KEGG pathway database

There was an up-regulation of several genes involved in ER-associated degradation (ERAD, ko04141) and the ubiquitin-proteasome system (UPS) after 24 h of hypo-saline stress in the adults, while many of the same genes were down-regulated under acute (1 h) salinity stress (Fig. 2; Additional file 4: Table S3). The ER pathway involves several processes, including: protein folding and translocation into the ER lumen, degradation of misfolded proteins through the ERAD system and proteolysis through the UPS. Amongst the genes up-regulated after 24 h were coral homologues of genes responsible for translocation into the ER lumen; the oligosaccharyl transferase (OST) and SEC61 protein transport systems. Genes involved in protein glycosylation also showed increased expression after 24 h; for example, glucosidase II (GlcII) increased by 0.66 log_2_FC (FDR 8.95E-06, Additional file 4: Table S3), and UDP-glucose/glycoprotein glucosyltransferase (UGGT) increased by 0.59 log_2_FC (FDR 8.82E-04). Moreover, luminal chaperones and co-chaperones were also up-regulated at 24 h, including the HSP70 family member GRP70, also known as binding immunoglobulin protein (BiP; 1.2.4351.m1; 1.3 log_2_FC; FDR 8.64E-19 at 24 h), along with the BiP co-chaperones ERdj1, ERdj3 and ERdj6 (DnaJ Hsp40 family members; 1.2.7940.m1, 1.2.25530.m1, 1.2.21656.m1). Increased expression was also observed for members of the ERAD retrotranslocon complexes, including the endoplasmic reticulum lectin 1 (XTP3B, 1.2.21359.m1), heat shock protein 90 kDa (GRP94, 1.2.15211.m1), translocating chain-associated membrane protein (TRAM, 1.2.11248.m1), and the translocon-associated protein (TRAP, 1.2.3165.m1).

Increased expression of components of the unfolded protein response (UPR) system was recorded after 1 and 24 h of hypo-saline stress (Fig. 2; Additional file 4: Table S3). The UPR system relies on three major transmembrane proteins involved in sensing stress that were differentially regulated during this experiment. Coral genes homologous to the serine/threonine-protein kinase/endoribonuclease IRE1 (IRE1), and its interacting pro-apoptotic effector BAX, were up-regulated after 1 h (0.30 log_2_FC, FDR 2.59E-02 and 0.41 log_2_FC, FDR 2.78E-02 respectively; Fig. 2; Additional file 4: Table S3). In addition, genes homologous to the eukaryotic translation initiation factor 2-alpha kinase (PERK), and the activating transcription factor 6 (ATF6) were up-regulated after 24 h of stress (0.60 log_2_FC, FDR 6.25E-03 and 0.42 log_2_FC, FDR 1.89E -02 respectively; Fig. 2; Additional file 4: Table S3).

### The response of genes involved in oxidative stress and osmoregulation

**Fig. 3:**
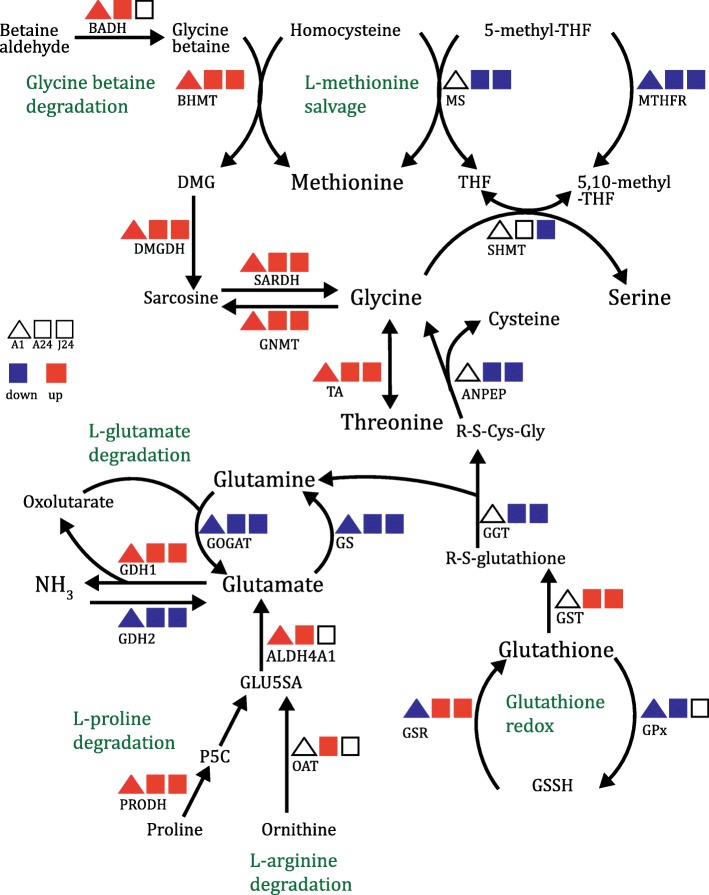
Expression of *A. millepora* homologues of genes involved in amino acid metabolism during hypo-osmotic stress in adult and juvenile corals. Colours represent up (red) and down-regulated (blue) genes (FDR < 0.05) after 1 h (triangle) in the adults (A1) and 24 h (squares) in the adults (A24) and juveniles (J24). Additional file 4: Table S5, provides the complete list of genes involved in this pathway and details of expression levels

Genes involved in the peroxisomal antioxidant system that showed increased expression after 24 h of hypo-saline stress include: two superoxide dismutases (SOD, by 0.41 and 0.43 log_2_FC; FDR 1.07E-02 and 4.68E-02), two catalases (CAT, by 0.49 and 1.44 log_2_FC; FDR 1.93E-31 and 1.96E-02), and five glutathione S-transferases (GST, EC:2.5.1.18) (Additional file 4: Table S3, S4). The glutathione (GSH) redox system also plays an important role in protection against oxidative damage. It comprises the enzymes glutathione peroxidase (GPx, EC 1.11.1.9) (oxidizing GSH to glutathione disulphide (GSSG)), and glutathione reductase (GSR) (reducing GSSG back to glutathione). During hypo-saline stress, the GSR homologue was up-regulated after 24 h, while the GPx homologue was down-regulated after 1 and 24 h of stress by − 0.38 and − 1.08 log_2_FC respectively (FDR 1.30E-03 and 7.61E-10; Fig. 3; Additional file 4: Table S5), indicating a balance towards GSH reduction.

Osmotic stress involves changes in the cellular concentrations of many inorganic and organic molecules, and this was corroborated by altered expression of many genes associated with transport of ions or organic molecules, including several solute carrier (SLC) families, ATPases, voltage-gated K^+^ channels, and voltage-dependant Ca^2+^ channels (VDCC). After 1 h of salinity stress, three of the nine Na^+^/(Ca^2+^ − K^+^) exchangers (SLC24) identified were up-regulated, while four Na^+^ and Cl^−^ dependent transporters (SLC6) were down-regulated (Additional file 4: Table S6). After 24 h, eight SLC6 genes and three SLC24 genes were down-regulated. In the case of ATPases, five genes were down-regulated after 1 h, whereas five were up-regulated after 24 h of stress. Amongst the ATPases, the relative expression of the sarco/endoplasmic reticulum Ca^2+^ ATPase (SERCA; an ER-associated Ca^2+^ influx channel) changed from − 1.40 log_2_FC at 1 h to 1.63 log_2_FC after 24 h (FDR 1.15E-09 and 3.59E-05 respectively). Conversely, expression of inositol 1,4,5-trisphosphate receptors (IP3Rs), which are Ca^2+^ efflux channel components, was down-regulated after 24 h (FDR − 1.35 log_2_FC; 4.05E-03; Fig. 2; Additional file 4: Table S3). In addition, three voltage-dependant Ca^2+^ channels were not differentially expressed after 1 h, but down-regulated after 24 h.

### Glycine betaine and glutamate catabolism under hypo-saline stress

GO analysis revealed an over-representation of terms associated with amino acid metabolism, with a strong response of genes implicated in glycine betaine catabolism following osmotic stress in the adults (Fig. 3; Additional file 4: Table S5). The first step in glycine betaine catabolism involves betaine-homocysteine *S*-methyltransferase (BHMT), which transfers a methyl group from glycine betaine to homocysteine to produce dimethylglycine (DMG) and methionine. In the work described here, two betaine-homocysteine *S*-methyltransferase (BHMT) homologues were up-regulated (by 2.5 and 5.43 log2FC; FDR 6.39E-70 and 2.38E-69) after 24 h of stress. The DMG produced by the BHMT reaction can be converted to glycine by two enzymes (DMGDH and SARDH, Fig. 3), homologues of both of which were up-regulated after 1 and 24 h of hypo-saline stress.

Hypo-saline stress also caused changes in the expression of genes involved in ammonia assimilation. The coral NADH-dependant glutamate dehydrogenase (GDH1), which catalyses the release of ammonia from glutamate, was up-regulated after 1 and 24 h of stress (log_2_FC of 0.47 and 2.54 respectively; FDR 1.21E-02 and 4.28E-55). Conversely, genes involved in ammonia assimilation - the NADPH-dependant GDH (GDH2), glutamine synthase (GS), and glutamate synthase (GOGAT) - were down-regulated (Fig. 3; Additional file 4: Table S5). Genes involved in the L-arginine degradation pathway were also up-regulated in hypo osmotic stress, expression of both ornithine transaminase (OAT), and pyrroline-5-carboxylate dehydrogenase (ALDH4A1) increasing (by 0.52 and 1.51 log_2_FC respectively; FDR 2.16E-05 and 1.10E-11) after 24 h (Fig. 3; Additional file 4: Table S5).

### The responses of coral juveniles to hypo-saline stress

The responses of adult and juvenile corals were similar after 24 h of stress for a substantial number of DEGs (1191) (Additional file 3: Figure S3). For example, genes encoding proteasome subunits, components of the UPR system, and glycine betaine catabolism were up-regulated in both juveniles and adults after 24 h (see above). Conversely, three important ER luminal chaperones (BiP, GRP94 and NEF) showed opposite expression trends in the two life stages, being up-regulated in adults but down-regulated in juveniles (Additional file 4: Table S3). Of the four treatments studied, the prolonged (48 h) exposure of juveniles resulted in the lowest number (1485, FDR < 0.05) of differentially expressed genes in response to hypo-saline conditions. After 48 h, expression levels of many genes that were differentially expressed after 24 h in juveniles had returned to control levels, suggesting that a degree of acclimation may have occurred. For example, only two ubiquitin-proteasome system (UPS) subunits were differentially expressed at 48 h, whereas ten were so at 24 h (Additional file 4: Table S3). A similar decrease was observed in the case of E2 ubiquitin-conjugation enzymes - from 13 to three up-regulated members after 24 and 48 h respectively (Additional file 4: Table S3).

## Discussion

Gene expression data revealed a strong response of the coral *A. millepora* to hypo-saline stress, with clear differences between acute salinity shock (1 h) and more prolonged (24 h) exposure in adult corals. Here we describe a group of genes that are part of a general response to stress in corals, and a second group that are known to respond to osmotic stress in other organisms but were not previously described in corals. The first group includes genes involved in antioxidant production, and in protein homeostasis (comprising molecular chaperones, components of the ER associated protein degradation (ERAD) and unfolded protein response (UPR) systems). The second group comprises genes involved in osmoregulation, including molecular transporters and amino acid metabolism, particularly glycine betaine. Together, variations in the expression of these two groups of genes provide insights into the molecular basis of hypo-osmotic stress in corals and the changes involved in adjusting to this stress over time.

### The common response to stress in corals

Hypo-saline stress induces expression of antioxidant defences that are protective against the reactive oxygen species (ROS) that arise in corals (and other organisms) as a result of a range of environmental stressors [40]. In the present study components of the general antioxidant repertoire of corals (catalases, superoxide dismutases and thioredoxin) that respond to thermal and to elevated CO_2_ stress [14, 24, 25, 41] were also found to respond to hypo-saline stress (Table 1). A second group of genes involved in general stress responses is the HSP family. For some time, HSPs have been investigated in the context of responses of corals to thermal stress [42–44], but the HSP repertoire has only recently been properly described in *A. millepora*, allowing comprehensive analyses of the response of this complex gene family to stress [24]. Whereas multiple HSP90 and HSP70 variants are present in corals, some of which can respond to a range of stressors [14, 25, 42, 45], specific HSPs appear to respond to most types of stress. For example, Moya et al. [24, 46] identified a specific *A. millepora* HSP70 that also responded to high CO_2_ and whose *A. hyacinthus* orthologue was involved in thermal tolerance [47]. Consistent with a role in the general stress response, this same HSP70 responded to hypo-saline conditions in the present study (1.2.19257.m1, 2.54 log_2_FC; FDR 3.42E-10 at 24 h, Table 1).

**Table 1:**
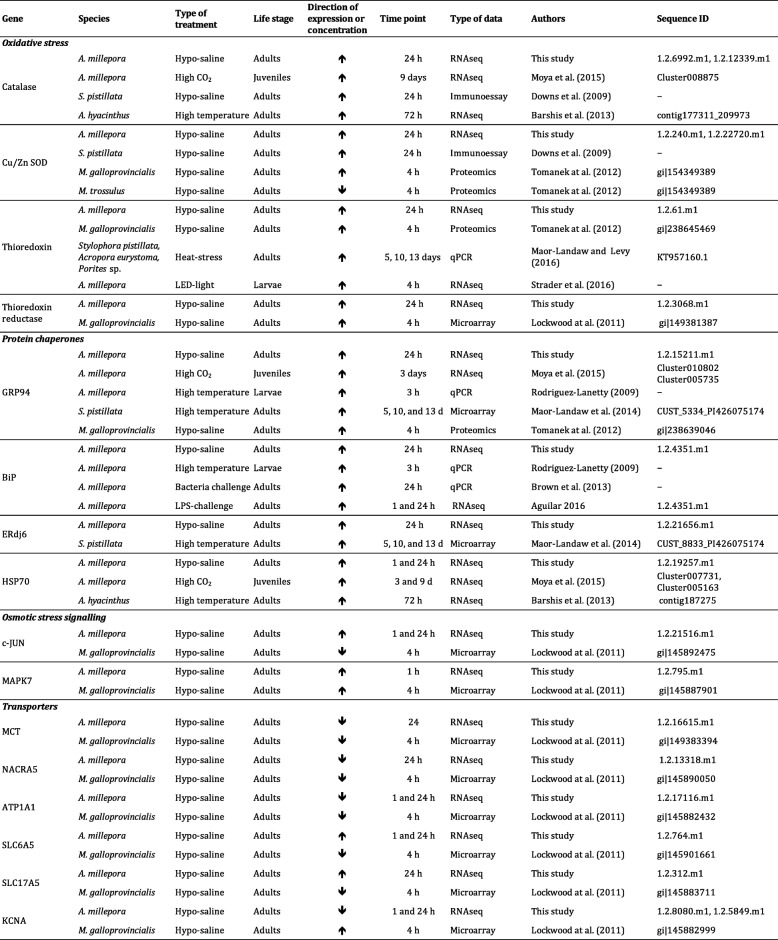
Comparison between genes that are differentially expressed in *A*. *millepora* adults under hyposaline conditions and other published gene expression or proteomic studies

Of the HSPs associated with ER processes, the luminal chaperone GRP94 (Fig. 2, Additional file 4: Table S3, 1.2.15211.m1) is of particular interest. As in other systems, this calcium-binding protein blocks apoptosis, and plays a key role in facilitating recovery from ER stress [48]. GRP94 expression was elevated after 24 h of salinity stress, and also responded to acute CO_2_ [24] and thermal stress in corals [43]. The mussel (*Mytilus galloprovincialis)* orthologue also responded to hypo-saline stress [49] (Table 1). In the present study, the ER-lumenal HSP70 BiP, which is involved in protein folding and is a component of the ERAD system [50] was up-regulated under hypo-saline conditions, and is also induced by challenge with bacteria [51] or lipopolysaccharide (LPS [52];). However, BiP was not differentially expressed under high CO_2_ stress [24], suggesting that it has a broad, but not universal, role in coral stress responses (Table 1).

Down-regulation of ERAD system components was observed after the acute salinity treatment (1 h); however unfolded protein response (UPR) system components were up-regulated after both 1 and 24 h, suggesting that misfolded proteins accumulated as early as 1 h after the onset of osmotic stress. The activation of the UPR system can have two opposite outcomes: it can promote survival and resistance to ER stress and/or it can activate a cell death response [53]. For example, in mammals the endoribonuclease inositol-requiring enzyme–1 (IRE1) signalling protein can interact with the pro-apoptotic protein BAX, or it can activate c-JUN to promote cell survival [53]. Like its mammalian orthologue, coral BAX promotes cell death [54], but up-regulation of BAX under hypo-osmotic stress was small compared to that of the pro-survival protein, c-JUN, suggesting that the latter outcome might predominate during hypo-saline stress. Previous studies by Maor-Landaw et al. [55] in *Stylophora pistillata* found that PERK increased during temperature stress, and expression of c-JUN and MAPK7 homologues increased under hypo-saline stress in mussel [21]. There is also evidence of a general increase of UPR associated genes in an expression module of the coral *Acropora hyacinthus* under high heat stress [56]. However, the present study is the first to document differential expression of the three main transmembrane proteins that regulate the UPR (BAX, IRE1, and PERK), and components of the corresponding downstream signalling pathways (Fig. 2; Additional file 4: Table S3).

### The specific response to hypo-saline stress in coral —Osmoregulation and transporters

As adjustments to hypo-saline conditions require cell volume regulation, transport of ions through membranes plays an important role in adjusting this osmotic potential, and is mediated by H^+^ translocating ATPases, Ca^2+^-ATPases, secondary active transporters, and channels [57]. While ion transport proteins have been extensively characterized in higher animals, fungi and plants [58, 59], little is known about these genes families in cnidarians (but see [60, 61]). When the results of the present study were compared with those derived from mussels under hypo-saline stress, the expression of several specific transporters (MCT, Nacra5, and ATP1A1) showed similar trends, whereas an opposite response was observed for others (SLC6A5, SLC17A5, and KCNA, Table 1). However, some of these apparent differences may be a consequence of the difficulty in identifying true orthologues across the deep evolutionary divide between molluscs and cnidarians (Table 1 and Additional file 4: Table S7). In general, and as mentioned by Lockwood and Somero [21], the responses of these transporters reflect two opposite adaptive mechanisms to stress: (i) moving ions across the membrane to stop cell swelling, and (ii) arresting the transport activities when solute concentrations inside the cell exceed requirements [62, 63]. Some of the results presented here might reflect these opposing activities, but also highlight the complexity of the gene families involved.

Marine invertebrates adjust their osmotic concentration not only by inorganic ion fluxes, but also via organic osmolytes such as taurine or betaines. Glycine betaine is thought to be an important osmolyte in corals, constituting > 90% of the organic solutes measured in *Fungia, Pocillopora, Montipora* and *Tubastrea* [18, 64]. Increased transcription of genes involved in glycine betaine catabolism was observed in the present study, implying that degradation of this compound occurred during hypo-osmotic stress (Fig. 3, Additional file 4: Table S5). Previous experiments on the effects of hypo-saline stress in the Pacific oyster *Crassostrea gigas* also found an increase in transcription of betaine-homocysteine S-methyltransferase (BHMT), a key enzyme of glycine betaine catabolism [65]. Glycine betaine concentrations decrease under hypo-saline stress in the marine alga *Platymonas subcordiformis* [66], consistent with this compound acting as an osmoticum. Interestingly, the concentration of the organic sulfur compound dimethylsulfoniopropionate (DMSP), a well-known osmolyte in plants which increases in corals under heat stress [28], also increased under hypo-saline conditions during our study [30], suggesting that DMSP is unlikely to act as an osmolyte in corals, but rather as a scavenger of ROS and molecular sink for excess methionine.

In a range of marine invertebrates, including the sea anemone *Metridium senile* and the bivalve *Noetia ponderosa* [15, 16], free amino acid (FAA) levels also decrease in response to hypo-osmotic stress. However, the limited body of work on FAA metabolism in corals is not consistent with this trend (as it was found to increase in the coral *A. aspera* during hypo-saline stress [19]). The data presented here suggest that amino acid catabolism increased under hypo-saline stress, leading to increased ammonia production (GDH up-regulated, Fig. 3), but measurements of amino acid levels are needed to confirm osmolyte responses under hypo-saline stress.

### The response of adult coral vs. juveniles to hypo-saline stress

Whereas previous work on salinity stress has focused on adult corals, this is the first investigation to consider both adult and juvenile corals. Since the juveniles were aposymbiotic, the response of juveniles documented here permits insights into molecular mechanisms operating in the coral animal in the absence of metabolic contributions [67] from endosymbiotic Symbiodiniaceae. Therefore, an important caveat in interpreting these results is that the presence/absence of endosymbiont in adults/juveniles. Despite this difference, after 24 h of osmotic stress, many aspects of the response were common between the adults and juveniles - for example, genes involved in adjusting cell volume (e.g., transporters, betaine catabolism). Several genes involved in amino acid metabolism also responded in the same way in both juveniles and adults (Fig. 3), and of particular interest was the increase of BHMT, an enzyme involved in methionine biosynthesis, at both time points, with a maximum change after 48 h in the juveniles (Additional file 4: Table S5; 4.04 Log_2_FC; FDR 0). This observation could be linked to increases in DMSP production observed with both life history stages during this experiment [30], since methionine is a precursor of DMSP. By contrast, the antioxidant system was up-regulated in adults and largely unaffected by hypo-osmotic stress in juveniles (Additional file 4: Table S4). This result could be explained as the adult corals have evolved mechanisms to withstand ROS produced by their endosymbionts [68].

In the case of juveniles, significantly fewer genes were differentially expressed after 48 h compared to 24 h of exposure to hypo-saline conditions, with the return to baseline levels of many of the genes implicated in proteolysis and osmoregulation suggesting a degree of acclimation had occurred after 48 h. A precedent for this is provided by the work of Moya et al. [24] on the response of *A. millepora* juveniles to elevated CO_2_, where short term (3 d) exposure to elevated CO_2_ caused changes in the expression of multiple genes, most of which returned to baseline levels after 9 d of exposure. Whilst these results suggest the possibility of acclimation to hypo-saline stress after 48 h, experiments with longer exposure times are needed to understand if this response is maintained and how such treatments impact coral physiology.

## Conclusions

During extreme floods, such as occurred on the GBR in 2010–2011, shallow reefs (< 5 m) were exposed to salinity below 25 PSU for up to 15 days [12]. The frequency and severity of heavy rainfall events are predicted to increase by 2050 [69, 70], leading to corresponding increases in the exposure of adult and juvenile corals to hypo-saline conditions. The data presented here highlight specific pathways involved in the molecular response of corals to salinity stress, and imply that juvenile corals may have the ability to adjust to hypo-saline conditions during heavy rainfall events. However, longer term experiments, combined with physiological and proteomic analyses, should be a high priority.

## Additional files

Additional file 1 **Figure S1.** Principal component analysis (PCA) from the normalized expression values of 26,622 genes in coral adults and juveniles. (a) Adults, each colour represents a colony (C1-C5, *n* = 4 per colony). (b) Juveniles, each colour represents a salinity treatment (*n* = 11 per treatment). PCA was generated from the *variance stabilizing transformation (VST) values* using “ggplot” in R [33]. (PDF 118 kb). (PDF 239 kb)

Additional file 2 **Figure S2.** Total number of differentially expressed genes (DEGs) (FDR < 0.05) for each dataset. With the corresponding number of up-regulated (red) and down-regulated (blue) genes. (PDF 54 kb) (PNG 52 kb)

Additional file 3 **Figure S3.** Venn diagrams of the differentially expressed genes (FDR < 0.05) after 24 h hypo-saline stress that were up- (red) and down- (blue) regulated in the adults and juveniles *A. millepora* corals. Indicating the subset of shared genes between each set of expression. (PDF 376 kb) (JPG 367 kb)

Additional file 4 **Table S1.** Differentially expressed genes and their GO as in the heat map Fig. 1. **Table S2.** Gene enrichment analysis from BiNGO in Cytoscape 3.1.1. Each gene set identifies numbers of differentially expressed genes (FDR < 0.05) in GO categories responding to salinity stress id adults or juveniles at the specified time points. **Table S3.** *A. millepora* homologues to the ER protein processing system. Results of the KEGG protein processing in the ER (nve04141) pathway searched in the *A. millepora* protein predictions. Log_2_FC values of significantly expressed (FDR < 0.05) genes in response to the treatment (hypo-saline) over the control (35 PSU). **Table S4**. *A. millepora* homologues to the peroxisome and lysosome systems. Log_2_FC are values of significantly expressed (FDR < 0.05) genes in response to the treatment (hypo-saline) over the control (35 PSU). **Table S5** *A. millepora* homologues to amino acids metabolism. Log_2_FC are values of significantly expressed (FDR < 0.05) genes in response to the treatment (hypo-saline) over the control (35 PSU). **Table S6.** *A. millepora* homologues to membrane transporter. Log_2_FC are values of significantly expressed (FDR < 0.05) genes in response to the treatment (hypo-saline) over the control (35 PSU). **Table S7.** Comparison between differentially express (FDR < 0.05) genes in *A. millepora* under hypo-saline conditions and other published gene expression or proteomics data. (XLSX 130 kb) (XLSX 118 kb)

## Abbreviations

ANPEP: Aminopeptidase
BADH: Betaine-aldehyde dehydrogenase
BHMT: Betaine-homocysteine methyltransferase
DMGDH: Dimethylglycine dehydrogenase
GDH1: Glutamate dehydrogenase (NADH)
GDH2: Glutamate dehydrogenase (NADPH)
GGT: Gamma-glutamyltranspeptidase
GNMT: Glycine N-methyltransferase
GOGAT: Glutamate synthase
GPx: Glutathione peroxidase
GS: Glutamine synthetase
GSR: Glutathione reductase
GST: Glutathione S-transferase
MS: Methionine synthase
MTHFR: Methylenetetrahydrofolate reductase
OAT: Ornithine--oxo-acid transaminase
PRODH: Proline dehydrogenase
PSU: Practical salinity units
SARDH: Sarcosine dehydrogenase
SHMT: Serine hydroxymethyltransferase
TA: Threonine aldolase
