# Supplementary figures and images for: Transcriptomic analysis reveals protein homeostasis breakdown in the coral Acropora millepora during hypo-saline stress
Source: BMC Genomics. 2019 Feb 20;20:148. doi: 10.1186/s12864-019-5527-2 (PMC6381741; doi:10.1186/s12864-019-5527-2)

a

Adult PC1 vs PC2

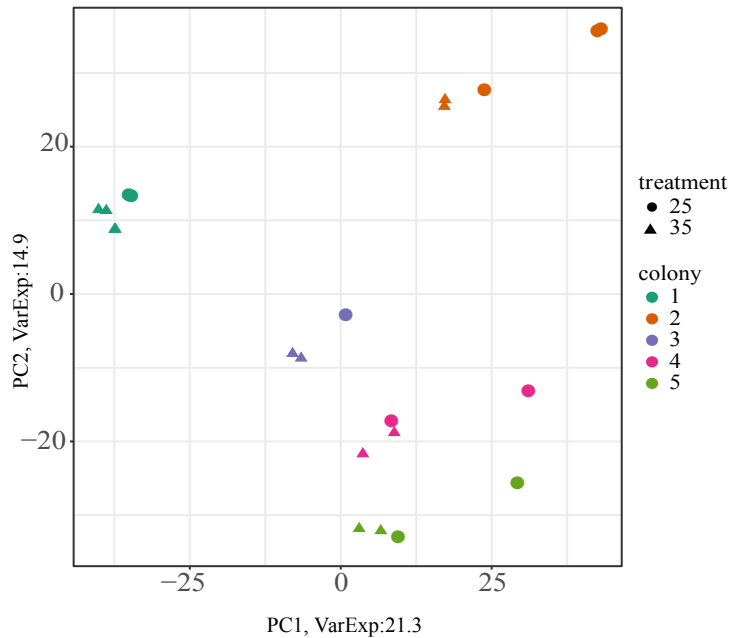

b

Juveniles PC1 vs PC2

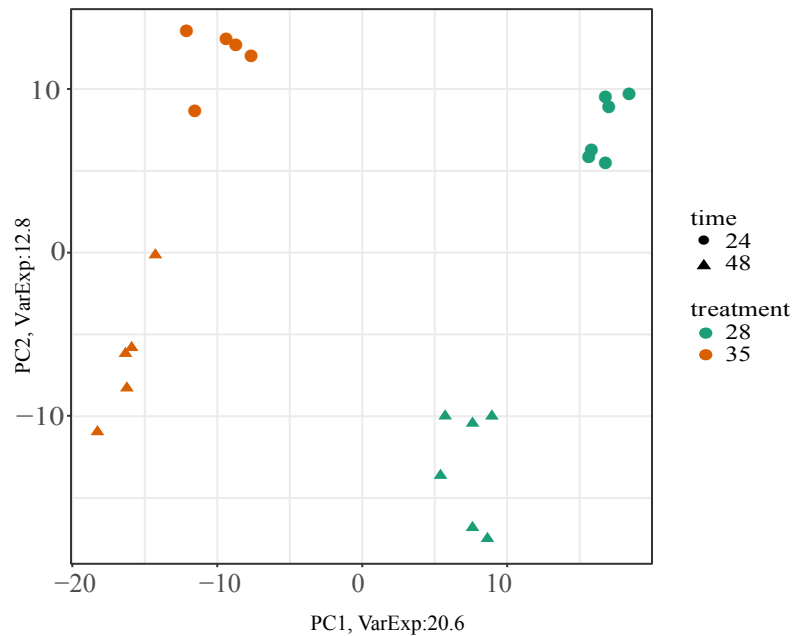

Supplement: Supplementary file 1 — Figure S1. Principal component analysis (PCA) from the normalized expression values of 26,622 genes in coral adults and juveniles. (a) Adults, each colour represents a colony (C1-C5, n = 4 per colony). (b) Juveniles, each colour represents a salinity treatment (n = 11 per treatment). PCA was generated from the variance stabilizing transformation (VST) values using “ggplot” in R [33]. (PDF 118 kb). (PDF 239 kb) [file 12864_2019_5527_MOESM1_ESM.pdf]

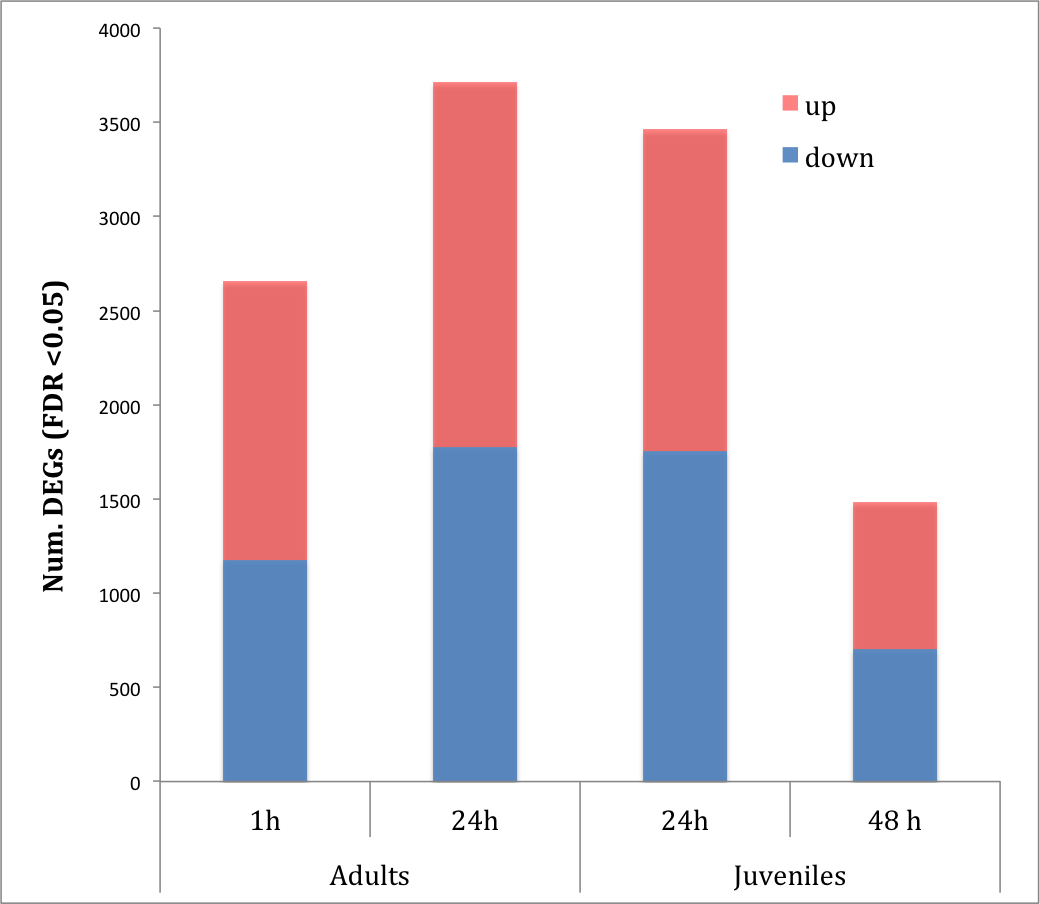

Supplement: Supplementary file 2 — Figure S2. Total number of differentially expressed genes (DEGs) (FDR < 0.05) for each dataset. With the corresponding number of up-regulated (red) and down-regulated (blue) genes. (PDF 54 kb) (PNG 52 kb) [file 12864_2019_5527_MOESM2_ESM.png]

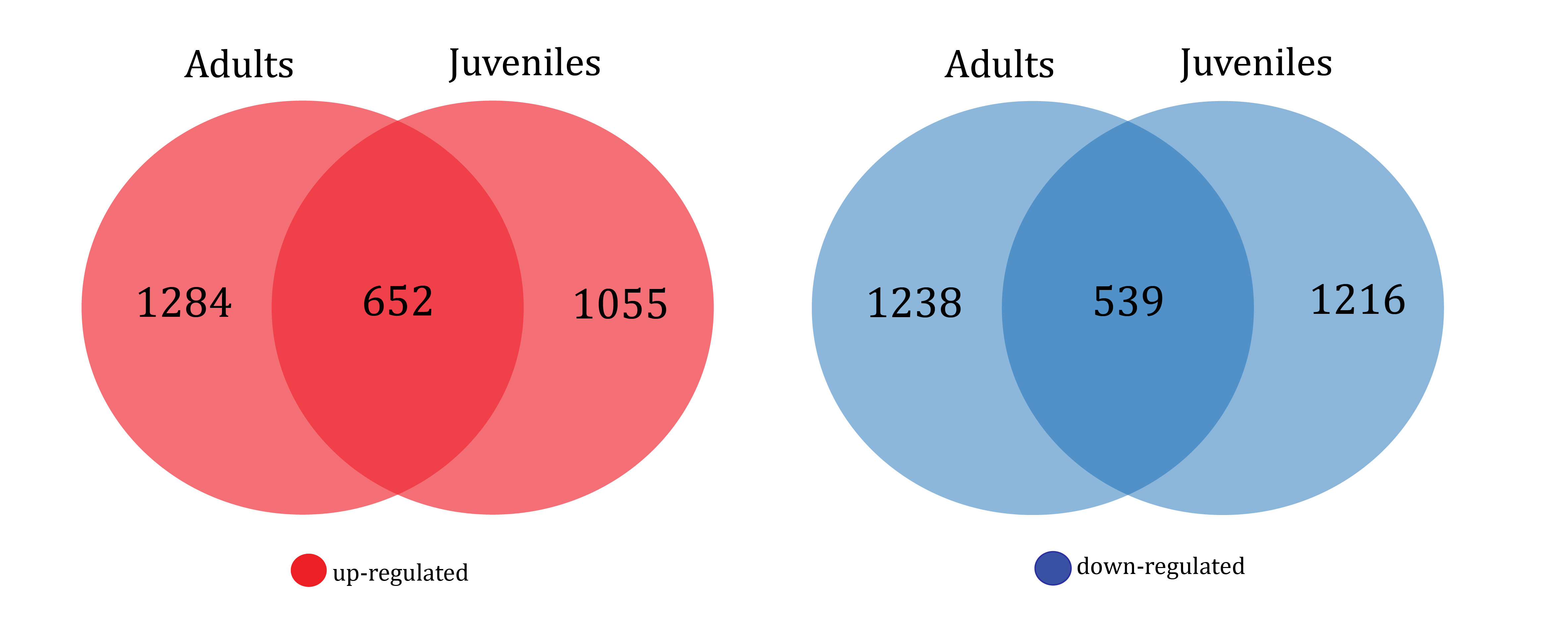

Supplement: Supplementary file 3 — Figure S3. Venn diagrams of the differentially expressed genes (FDR < 0.05) after 24 h hypo-saline stress that were up- (red) and down- (blue) regulated in the adults and juveniles A. millepora corals. Indicating the subset of shared genes between each set of expression. (PDF 376 kb) (JPG 367 kb) [file 12864_2019_5527_MOESM3_ESM.jpg]
